# Pharmacovigilance of artemether-lumefantrine in pregnant women followed until delivery in Rwanda

**DOI:** 10.1186/1475-2875-11-225

**Published:** 2012-07-06

**Authors:** Stephen Rulisa, Nadine Kaligirwa, Steven Agaba, Corine Karema, Petra F Mens, Peter J de Vries

**Affiliations:** 1 National University of Rwanda, University Teaching Hospital of Kigali, BP 655, Kigali, Rwanda; 2 Center for Infection and Immunity Amsterdam, Center for Poverty Related Communicable Diseases (CPCD)Academic Medical Center, Meibergdreef 9, 1105 AZ, Amsterdam, The Netherlands; 3 Rwanda Biomedical Centre, Center for Treatment and Research on AIDS, Malaria and TB (TRAC-PLUS), BP 2717, Kigali, Rwanda; 4 Academic Medical Center, Division of Infectious Diseases, Tropical Medicine and AIDS, Meibergdreef 39, 1105 AZ, Amsterdam, The Netherlands; 5 Royal Tropical Institute/Koninklijk Instituutvoor de Tropen (KIT), KIT Biomedical Research, Meibergdreef 39, 1105 AZ, Amsterdam, The Netherlands

## Abstract

**Background:**

The World Health Organization presently recommends Artemisinin-based combination therapy (ACT) as first-line therapy for uncomplicated *P. falciparum* malaria. Many malaria-endemic countries, including Rwanda, have adopted these treatment guidelines. The Artemisinin derivative Artemether, in combination with lumefantrine, is currently used in Rwanda for malaria during the second and third trimesters of pregnancy. Safety data on the use of ACT in pregnancy are still limited though and more data are needed.

**Methods:**

In this pharmacovigilance study, the exposed group (pregnant women with malaria given artemether-lumefantrine), and a matched non-exposed group (pregnant women without malaria and no exposure to artemether-lumefantrine) were followed until delivery. Data were collected at public health centres all over Rwanda during acute malaria, routine antenatal visits, after hospital delivery or within 48 hours after home delivery. Information gathered from patients included routine antenatal and peri-partum data, pregnancy outcomes (abortions, stillbirths, at term delivery), congenital malformations and other adverse events through history taking and physical examination of both mothers and newborns.

**Results:**

The outcomes for the total sample of 2,050 women were for the treatment (n = 1,072) and control groups (n = 978) respectively: abortions: 1.3% and 0.4%; peri-natal mortality 3.7% and 2.8%; stillbirth 2.9% and 2.4%; neonatal death [less than or equal to]7 days after birth 0.5% and 0.4%; premature delivery 0.7% and 0.3%; congenital malformations 0.3% and 0.3%. A total of 129 obstetric adverse events in 127 subjects were reported (7.3% in the treatment group, 5.0% in the control group). In a multivariate regression model, obstetric complications were more frequent in the treatment group (OR (95% CI): 1.38 (0.95, 2.01)), and in primigravidae (OR (95% CI) 2.65 (1.71, 4.12) and at higher age (OR per year: 1.05 (1.01-1.09).

**Conclusions:**

There were no specific safety concerns related to artemether-lumefantrine treatment for uncomplicated falciparum malaria in pregnancy. However, more obstetric complications were observed in the treatment group. These increased occurrence of complications could, however, be caused by the malaria episode itself, but further assessment is required.

## Background

Malaria poses a heavy burden of disease in many countries, especially in Africa, and contributes to at least 40% of all consultations in health centres in Rwanda [1]. In response to rising mortality from malaria, linked to increasing parasite resistance and a decrease in the efficacy of older anti-malarials, such as chloroquine and sulphadoxine-pyrimethamine (SP), the World Health Organization (WHO) now recommends the use of Artemisinin-based combination therapy (ACT) as first-line treatment of uncomplicated *P. falciparum* malaria [2]. Many countries, including Rwanda, have shifted their malaria treatment guidelines to using ACT as the first choice, including for pregnant women in their 2nd and 3rd trimester [3]. Artemether-lumefantrine (AL; Coartem®, Novartis) is an ACT that is very effective and well-tolerated [4,5]. Despite the fact that AL is not registered for use during pregnancy, WHO Treatment Guidelines recommend that, in the second and third trimesters of pregnancy, only an ACT that is known to be effective in that particular country/region, should be used [2,6]. In the first trimester, an ACT should only be used if there is no other treatment immediately available. The limited safety data continue to restrict ACT use in the first trimester, but the rapid adoption of ACT means that they may be prescribed inadvertently in early unsuspected pregnancies [2]. Uncertainty about the use of anti-malarials in pregnancy may lead to loss of public confidence in a drug and poor adherence to the treatment regimen. This may ultimately lead to treatment failure and the development of parasite resistance [2]. Further studies are required in women in all stages of pregnancy to effectively assess the risk-benefit profile of Artemisinin compounds [7].

Stringent guidelines discourage pharmaceutical companies from including pregnant women or even women of child-bearing potential in all stages of clinical trials [8]. Current guidelines for the inclusion of pregnant women in controlled trials vary from a cautious attitude of the US Food and Drug Administration (FDA) [8] to advocating the inclusion of pregnant women by *the Joint United Nations Programme on HIV/AIDS (UNAIDS)* (the Joint United Nations Programme on HIV/AIDS) and WHO [9].

Often, only non-clinical data are available for assessing the effects of anti-malarials during pregnancy [10]. Animal studies have shown adverse effects of the Artemisinin derivatives on early foetal development [11,12] and they have only partly been evaluated during early pregnancy in humans [13]. A further challenge is to distinguish the potential effects of ACT from the effects of malaria in pregnancy, which include maternal anaemia, low birth weight, adverse effects on foetal development and, in low transmission areas, an increased risk of severe malaria [2]. Active monitoring of anti-malarials in pregnancy is recommended by WHO and the European Medicines Agency (EMA) [2,10]. The WHO recommends that Pregnancy Exposure Registries be used [2], as these can monitor and record data on drug exposure and outcomes in pregnancy and may provide reassurance on the potential risks associated with anti-malarial drugs such as AL [14].

Unfortunately, the health infrastructure in many malaria endemic areas does not allow to consistently collecting data on drug-related adverse effects and data cannot be extracted from the established passive pharmacovigilance systems in industrialized countries where tropical diseases are rare and treatment and population characteristics may be different (genetics, access to healthcare, socio-economic conditions). An international anti-malarial exposure registry has been created to follow the effects of implementation of ACT in Africa; however, it has not yet been analysed for data on anti-malarials in pregnancy.

In this prospective observational study in Rwanda, a group of urban and rural pregnant women receiving AL for malaria and pregnant women without malaria and who were not exposed to AL were closely followed to determine whether adverse outcomes were more frequent after administration of AL for malaria.

## Methods

### Study design

This cohort study took place from June 2007–July 2009 at ten health centres in Rwanda. The treatment group consisted of women who were prescribed AL for an episode of malaria in their second and third trimester of pregnancy, according to the national Rwandan guidelines for treatment of malaria. Women could be included immediately after the decision to treat with AL for malaria had been made, hereafter called “prospective” inclusion. Women who, during antenatal clinic attendance, were found to have been treated with AL during that pregnancy could also be included “retrospectively” if treatment could be verified from the patient prescription and treatment register at the health centre. The unexposed group consisted of pregnant women with no history of previous or current treatment with AL in the existing pregnancy and without any signs or symptoms of malaria. In order to estimate gestational age last menstruation, fundal height and date of quickening were recorded and correlation of at least two of the three factors was used for the age determination. Data were collected from the following participating primary health centres and district hospitals, located in malaria-endemic regions: Muhima (Kigali), Munyaga, Rwamagana and Rubona (Rwamagana district), Rukara (Kayonza district), Bukora (Kirehe district), Karambi (Ruhango District), Busoro (Nyanza District), Mashesha (Rusizi District) and Mubuga (Karongi District). All health centres provided antenatal care for pregnant women. Data were also collected from district hospitals where study subjects were referred to for delivery. The study was approved by the Rwandan National Ethics Committee prior to commencement. Each patient gave written informed consent before entry into the study.

### Study population

#### Exposed cohort women with malaria treated with AL

Pregnant women above the age of 18 years were included in the study if the woman was to be treated with AL after diagnosis of simple (uncomplicated) P. falciparum malaria. The diagnosis of malaria was established through a positive blood smear or based on clinical symptoms. Patients of presumed malaria, i.e. when signs and symptoms were suggestive of malaria and were accordingly treated with AL but in the absence of microscopy or rapid diagnostic tests (RDTs), were also included. Pregnancy was detected clinically or by a beta human chorionic gonadotrophin pregnancy urine test. Gestational age was verified by ultrasound in a subset of subjects. This study also tried to capture inadvertent AL treatment during the first trimester by retrospective inclusion.

#### Non-exposed cohort women without malaria treatment with AL

Following recruitment of a patient in the treatment group, a woman with a similar stage of pregnancy and without history of previous or current treatment with AL in the existing pregnancy was selected at the same health centre during routine attendance at the antenatal clinic and invited to participate in the study as part of the control group. These control women were confirmed as at the moment of enrolment having no malaria by a negative blood smear. Previous malaria episodes that were treated with quinine instead of AL were considered as control women as well. Initially unexposed control mothers who developed malaria and were treated with AL at any point during follow-up were thereafter considered part of the treatment group.

### Study objectives and procedures

Data were collected from enrolled patients and control women upon recruitment at health centres through case report forms (CRFs). Baseline data included demographic data, number of pregnancies, gestational age, medical and obstetric examination, body temperature, malaria symptoms, usage of anti-malarials or other drugs and HIV serology. Malaria treatment response was classified as adequate clinical and parasitological response, late treatment failure (specifying late clinical and late parasitological failure) or early therapeutic failure, according to internationally accepted criteria [2].

All subjects were assigned a unique study number for recording. CRFs were filled out for exposed and non-exposed subjects during monthly antenatal clinic visits and upon any other visits to the health centre for health concerns, until delivery.

CRFs were updated with data during routine follow up after malaria or during antenatal clinic attendance, registering normal/abnormal development of the pregnancy and occurrence of symptoms suggestive of drug-related adverse events (AEs). CRFs were designed to capture adverse obstetric outcomes (abortion, peri-natal mortality, stillbirth, pre-term delivery, and unexplained neonatal death ≤7 days after birth), adverse infant outcomes (congenital malformations regardless of the pregnancy outcome, and neurological problems) and other AEs through history taking and physical examination of both mothers and newborns.

Serious AEs (SAEs), were those which resulted in death; were immediately life-threatening; resulted in persistent or significant disability/incapacity; resulted in a congenital anomaly/birth defect of the new born; required inpatient hospitalization or prolonged existing hospitalization or were deemed “important medical events” by the investigator. Serious adverse events had to be reported to the study coordinator within 24 hours after notification to the health centre.

The cause of all adverse pregnancy outcomes was investigated by structured questioning of the mother about pre-mortal symptoms to determine the likely cause of death. Women were encouraged to comment on suspected causes of these events.

Women in both groups were encouraged to deliver at the health centre with nurses’ or midwife’s aid, or at the district hospital, rather than to deliver at home. Women presenting to the hospital or health centre for delivery were asked about any complications experienced during the pregnancy. Following delivery, general health status was assessed in all newborns including scleral icterus, APGAR scores and prematurity. Women undergoing a home delivery were asked to bring in their newborn for medical evaluation at the health centre within 48 hours assisted by the community health care workers (not informed to which group the women belonged). A thorough physical examination was performed on the foetus by nurses/doctors trained to detect congenital defects and physical abnormalities. At follow up visits, a general assessment of the infant development (e.g. sitting unsupported, movement, lifting head) and possible defects in psychomotor and neurological development, using the WHO motor development milestones [15], was performed.

### Statistical analysis

A sample size of 1,000 treated and 1,000 untreated pregnant women was needed to detect a two-fold or greater difference in the relative risk of congenital malformations in the treatment group with 80% power and 95% confidence, based on the assumption that the incidence in the general population, and thus the un-exposed control group, is 2.5%.

Standard parametric statistical tests were used for comparison of normally distributed nominal and numeric data. Non-normally distributed data were analysed with the appropriate non-parametric tests. Normality was tested with the Kolmogorov-Smirnov test and by visual checks of frequency distributions and QQ-plots. Comparison of proportions between categorical and ordered categorical variables was done with Chi-square tests or Fisher’s exact tests. Statistical significance was determined at the 5% level (p < 0.05).

The associations between the main outcomes, obstetric complications and congenital malformations, were analysed in univariate and multivariate logistic regression models. Potential confounders, age of the mother, gravidity (primigravidae versus secundi- and multigravidae) and gestational age (as first trimester versus second and third trimester) were included in the model. Results were expressed as odds ratios.

## Results

This study recruited 2,070 pregnant women; two patients withdrew from the study early, and 18 patients had incomplete data sets and therefore were not considered during analysis, leaving 2,050 (99%) women: 1,072 pregnant women who were treated with AL for uncomplicated malaria and 978 women in the control group. Patient characteristics are shown in Table 1.

**Table 1:** Patient characteristics

**Inclusion**	**Study groups**		
	Pregnant women treated with AL for malaria (n = 1072)	Pregnant women without malaria (n = 978)	**P-value**
Followed prospectively at AL treatment	358 (33.4%%)	885 (90.5%)	
Recruited at second AL treatment during this pregnancy	6		
Recruited after recovery from malaria but before delivery	513 (47.9%%)	n/a	
Recruited after delivery	188 (17.5%)	93 (9.5%)	
Delivered during treatment	7 (0.7%)	n/a	
**Median age** (min–max, yrs)	26.0 (17–48)	27.0 (16–47)	0.091
**Gravidity**			0.002
Primigravidae	320 (29.9%)	210 (21.5%)	<0.001
Multigravidae	712 (66.4%)	730 (74.6%)	
Unknown	40 (3.7%)	38 (3.9%)	
Median gravidity (min–max)	2 (1–12)	3 (1–11)	<0.001
Median parity (min-max)	1 (0–10)	2 (0–9)	<0.001
Mean gestational age (95% CI, weeks)	25.8 (25.3, 26.4; n = 1052)	28.5 (28.0, 29.0; n = 973)	<0.001
**Trimester**			
First	96 (9.0%)	43 (4.4%)	<0.001
Second	434 (40.5%)	341 (34.9%)	
Third	542 (50.6%)	594 (60.7%)	
**Reported Medical History**			NS
1st AL treatment during this pregnancy >56 days before 2^nd^ treatment	4		
Quinine treatment during this pregnancy		6	
> 56 d before AL	1		
Just before delivery, > 56 after AL	1		
Intermittent Presumptive Treatment with SP	242 (23.4%; n = 1032)	265 (28.2%; n = 940)	0.016
Hypertension	0	1 (0.1%)	
Diabetes mellitus	1 (0.09%)	1 (0.1%)	

A total of 96 women (9.0% of the treatment group) inadvertently received AL during their first trimester of pregnancy and were included in the study when this was identified during routine obstetric consultations. Some women were also recruited retrospectively after confirmation of AL treatment for malaria from the treatment register and their prescription card and henceforth followed. Miscalculation of the gestational age at the time of treatment or unawareness of pregnancy was probably the cause of women being treated with AL during their first trimester of pregnancy.

### Pregnancy outcomes

The outcome of malaria treatment and obstetric outcome are shown in Table 2. Table 3 summarizes the observed congenital malformations. There were no statistically significant differences between any of the outcomes except for obstetric complications (p > 0.05). Overall peri-natal mortality was similar in both the treatment group and the control group (3.7% and 2.8%, respectively). Stillbirths occurred in 31 patients (2.9%) treated with AL and 23 control subjects (2.4%). Neonatal death within 7 days after birth was similar for both groups (0.7% in the treatment group and 0.4% in the control group). The number of infants who were delivered normally but then died during follow-up was 11 in the AL-treated group and 10 in the control group (detailed results will be reported separately). Three mothers who received AL treatment and one mother receiving no malaria treatment died after delivery.

**Table 2:** Malaria treatment and pregnancy outcomes

	**Study groups**		
	**Pregnant women**	**Pregnant women**	**P value**
**Treatment response**			
**Adequate clinical and parasitological**	**1068 (99.6%)**		
**Late treatment failure**	**4 (0.4%)**		
Parasite recurrence < 56 days after first AL treatment treated with quinine	**2**		
Parasite recurrence < 56 days after first AL treatment, treated again with AL	2		
**Obstetric outcome**			
**Uncomplicated at term deliveries**	**992 (92,5%)**	**926 (94.7%)**	
Mean birth weight of babies born at term	*3.20 (3.17, 3.24; n = 936)*	*3.22 (3.19, 3.25; n = 893)*	NS
Length (95% CI, cm)	*49.0 (48.6, 49.3; n = 666)*	*49.2 (49.0, 49.5; n = 645)*	NS
Head circumference (95% CI, cm)	*32.3 (32.1, 32.6; n = 644)*	*32.5 (32.3, 32.7; n = 632)*	NS
**Obstetric complications**	**80 in 78 (7.3%) 78 patients**	**49 in 49 (5.0%) subjects**	0.034
Abortion	*14 (1.3%)*	*4 (0.4%)*	
Still birth	*31 (2.9%)*	*23 (2.4%)*	
Premature delivery total	8 (0.7%)	3 (0.3%)	
At term delivery, neonatal death	5 (0.5%)	4 (0.4%)	
Cesarean section	3 (0.3%)		
Infant death	11(1.0%)	10 (1.0%)	
Fetal distress at birth	3(0.3%)	2 (0.2%)	
Neonatal infection	0	2 (0.2%)	
Stillbirth & hydrocephalus	*1(0.1%)*	-	
Twins, first with cerebral anoxia	*1(0.1%)*		
Still birth and maternal death	*1(0.1%)*		
At term delivery, maternal death	*2 (0.2%)*	*1 (0.1%)*	

**Table 3:** Infant outcomes

	**Study groups**		
	**Pregnant women treated with AL** **for malaria (n = 1072)**	**Pregnant women without AL treatment (n = 978)**	**P value**
Total congenital malformations	3 (0.3%)	3 (0.3%)	NS
Congenital aglossia	1 (0.09%)	0	
Spina bifida	0	1 (0.1%)	
Thoracic asymmetry	0	1 (0.1%)	
Congenital malformation of left foot	1 (0.09%)	0	
Hydrocephalus (stillbirth baby)	1(0.09%)	0	
Neurological disorder	0	1 (0.1%)	
Twins	9 (0.8%) (one still birth twin)	3 (0.3%)	NS
Fetal distress	3 (0.3%)	2(0.2%)	NS

The univariate and multivariate analyses for obstetric outcome are shown in Table 4. Potential confounders included in the model were trimester, gestational age, gravidity and age of the mother. The final multivariate model analysing the effects on the obstetric events included treatment group, gravidity, trimester and mother’s age as a covariate. In this model there was no significant effect of treatment group on the occurrence of obstetric complications. The final model showed that obstetric complications were more frequent in the treatment group and in primigravidae and at increasing age.

**Table 4:** Univariate and multivariate analysis of obstetric outcome

	**Frequency**		**Univariate analysis**			**Multivariate analysis**		
	Total no. of patients	No. of patients With adverse events with obstetric complications (total = 127)	OR	95% CI	P- Value	OR	95% CI	P-Value
Group								
Pregnant women & malaria & AL treatment	1030	78(7.3%)	1.48	1.03, 2.15	0.36	1.38	0.95, 2.01	0.09
Healthy pregnant women	937	49 (5.0%)						
Trimester								
First trimester	137	10 (7.3%)	1.15	0.59, 2.25	0.74	1.04	0.53, 2.05	0.913
2 &3^rd^ trimester	1830	117(6.4%)						
Gravidity								
Primigravidae	529	53(10.0%)	2.05	1.42, 2.97	<0.001	2.65	1.71, 4.12	<0.001
Multigravidae	1438	74(5.1%)						
Mothers age (covariate)						1.048	1.01, 1.09	0.009

Retrospective inclusion of women who had been treated with AL for malaria was also allowed. In order to cope with the possibility that abortions after treatment with AL were not reported and, therefore, not included in the study, analyses were also ran on the subset of patients and controls who were included prospectively.

The total number of congenital malformations (six) was considered too small to do multivariate analyses.

A summary of recorded infant outcomes is shown in Table 3. Congenital malformations occurred with a similar frequency in both. Congenital anomaly also had same frequency of one baby in each group. There was only one infant in the control group with neurological problems, and no reports of this in the AL-treated group. There were two babies born with infection at birth (conjunctivitis) in the control group and none in the treatment group. Three patients delivered their babies by caesarean section.

## Discussion

The results from this pharmacovigilance study in Rwanda showed a significantly increased frequency of obstetric adverse outcomes but no significant increase in congenital defects after AL treatment for malaria during the first second and third trimesters of pregnancy, compared with pregnant women who had no malaria and received no anti-malarial treatment. The significant over-expression of obstetric events was observed in the AL-treated group. The slightly higher rates of abortion, peri-natal mortality, stillbirth and premature delivery should probably be regarded as complications of acute malaria itself. However, the distinction between the effects of malaria and AL exposure could not be made in this study. Chronic malaria in pregnancy is associated with low birth weight, increased anaemia and adverse effects on foetal growth [2]. It is estimated that around 8–14% of low birth weight and 3–8% of infant mortality is related to pregnancy-associated malaria, which relates to a global annual estimate of 75,000–200,000 infant deaths attributable to malaria infection during pregnancy [16].

The information gathered from this study adds weight to the limited, but expanding, body of evidence on the use of AL in the second and third trimesters of pregnancy. The safety of Artemisinins in pregnant women has been the subject of a number of publications, and AL is the ACT with the most data on safety in pregnancy so far [2]. Several studies have reported that Artemether and AL do not have teratogenic effects [17-20]. A recent study in Zambia evaluated the safety of AL women with malaria in all trimesters of pregnancy [21], and exposure to AL was not linked to any particular safety risks with regards to perinatal mortality, birth defects, or developmental impairment. Patients receiving AL treatment during the first trimester of pregnancy had no greater risk of peri-natal or neonatal infant death or stillbirth [21].

Strengths and limitations of the study were both apparent. The sample size was large, and women were followed closely for any adverse event. In this study loss-to-follow-up was very low, besides the implementation of active follow-up by a study nurse this is partly because of the nature of the Rwandese health system which gives incentive to any pregnant women who completes all the antenatal visits during pregnancy like delivering for free at the health facility. In addition Rwanda has a nation-wide health insurance programme that covers over 95% of the population. The creation of a Rwandan anti-malarial exposure registry to follow the effects of implementation of ACT in pregnant women represents an important step forward in pharmacovigilance in this vulnerable population. Not all women had malaria confirmed via a positive blood smear and, therefore, it may be possible that women who were diagnosed based on clinical symptoms alone (presumed malaria) may have been suffering from another febrile condition. At the time of this study, malaria was classified and treated as either confirmed or presumed. This represented normal clinical practice in Rwanda, which has changed now; rapid diagnostic tests (RDTs) were only introduced in April 2008 [22]. Only patients with uncomplicated malaria in their second and third trimesters of pregnancy were enrolled in the study, as in national policy; however, subsequent analysis revealed that a small group of patients were actually in their first trimester during the study. This may be due to the fact that health care workers at the primary health centers may not be able to estimate the gestational age properly or even the women themselves do not report pregnancy accurately. This study does not include the follow-up of children for minor or undetectable neurological defects; follow-up of children will be reported in a different study.

Further evidence on the safety of pregnant women exposed to ACT during the first trimester of pregnancy is required. In general, there are ethical issues associated with the inclusion of women in randomized controlled trials in the first trimester of pregnancy. However, the WHO has suggested that there may be occasions where the need for effective treatment of the pregnant febrile patient may outweigh the potential for toxicity to the foetus [6]. For example, in cases of severe *P. falciparum* malaria, where maternal mortality is high and thus an investigation into the efficacy and safety of a new drug would be justified, or in instances of ineffective current treatments where a study comparing ACT and standard treatment for women with a recrudescent infection could provide important information on abortions and malformations if treatment was delivered and monitored during the first trimester, and second and third trimester exposure would provide critical information on the incidence of low birth weight and stillbirths [6].

## Conclusions

Overall, peri-natal mortality and the incidence of congenital malformations was low in the AL-treated and control groups. There was however a significant increase in the overall obstetric adverse events in the treatment group. This may however be related to malaria or other febrile illness rather than AL treatment in pregnant women.

Although AL is used for treatment of malaria in the second and third trimesters, a number of women are also exposed in the first trimester, hence close monitoring of women in all stages of pregnancy is still needed to determine the safety of AL, especially with a long follow-up until children reach school age, to be able to detect minor neurological adverse events.

## Competing interests

The authors declare that they have no competing interests.

## Authors’ contribution

SR: Conception and design of the study, supervised data collection, draft of manuscript, analysis of data and manuscript writing. NK: participated in data collection, coordination and data entry. SA: participated in data collection and data entry. CK: Protocol writing. PFM: Conception and design of the study, draft of manuscript, analysis of data and writing of the manuscript. PJD: Conception and design of the study, draft of manuscript, analysis of data and writing of the manuscript. All authors read and approved the final manuscript
